# A Web-Based, Respondent-Driven Sampling Survey Among Men Who Have Sex With Men (Kai Noi): Description of Methods and Characteristics

**DOI:** 10.2196/50812

**Published:** 2024-05-20

**Authors:** Samart Karuchit, Panupit Thiengtham, Suvimon Tanpradech, Watcharapol Srinor, Thitipong Yingyong, Thananda Naiwatanakul, Sanny Northbrook, Wolfgang Hladik

**Affiliations:** 1 Informatics Section Business Services Office US Centers for Disease Control and Prevention Nonthaburi Thailand; 2 Division of Epidemiology Department of Disease Control Ministry of Public Health Nonthaburi Thailand; 3 Division of Global HIV & TB US Centers for Disease Control and Prevention Nonthaburi Thailand; 4 Division of Global HIV & TB US Centers for Disease Control and Prevention Atlanta, GA United States

**Keywords:** online respondent-driven sampling, web-based respondent-driven sampling, virtual architecture, men who have sex with men, Thailand, MSM, Asia, Asian, gay, homosexual, homosexuality, sexual minority, sexual minorities, biobehavioral, surveillance, respondent driven sampling, survey, surveys, web app, web application, coding, PHP, web based, automation, automated, design, architecture, information system, information systems, online sampling, HIV, sexually transmitted infection, STI, sexually transmitted disease, STD, sexual transmission, sexually transmitted, RDS, webRDS

## Abstract

**Background:**

Thailand’s HIV epidemic is heavily concentrated among men who have sex with men (MSM), and surveillance efforts are mostly based on case surveillance and local biobehavioral surveys.

**Objective:**

We piloted Kai Noi, a web-based respondent-driven sampling (RDS) survey among MSM.

**Methods:**

We developed an application coded in PHP that facilitated all procedures and events typically used in an RDS office for use on the web, including e-coupon validation, eligibility screening, consent, interview, peer recruitment, e-coupon issuance, and compensation. All procedures were automated and e-coupon ID numbers were randomly generated. Participants’ phone numbers were the principal means to detect and prevent duplicate enrollment. Sampling took place across Thailand; residents of Bangkok were also invited to attend 1 of 10 clinics for an HIV-related blood draw with additional compensation.

**Results:**

Sampling took place from February to June 2022; seeds (21 at the start, 14 added later) were identified through banner ads, micromessaging, and in online chat rooms. Sampling reached all 6 regions and almost all provinces. Fraudulent (duplicate) enrollment using “borrowed” phone numbers was identified and led to the detection and invalidation of 318 survey records. A further 106 participants did not pass an attention filter question (asking recruits to select a specific categorical response) and were excluded from data analysis, leading to a final data set of 1643 valid participants. Only one record showed signs of straightlining (identical adjacent responses). None of the Bangkok respondents presented for a blood draw.

**Conclusions:**

We successfully developed an application to implement web-based RDS among MSM across Thailand. Measures to minimize, detect, and eliminate fraudulent survey enrollment are imperative in web-based surveys offering compensation. Efforts to improve biomarker uptake are needed to fully tap the potential of web-based sampling and data collection.

## Introduction

The HIV pandemic continues to be a public health threat, especially among key populations at high risk for HIV, including people who inject drugs, sex workers, transgender women, and men who have sex with men (MSM). Globally, key populations and their sex partners now account for more than half of all new HIV infections [1]. In Thailand, a country with a highly concentrated epidemic, MSM account for an estimated 12% of prevalent [2] HIV. Estimating the burden of HIV disease, risk, and access to and uptake of HIV-related services requires program and population-based survey data that are of high quality. Current standards for key population surveys include respondent-driven sampling (RDS) [3,4] and time-location sampling [5]. Both designs typically only allow for localized and urban surveys and, as with any survey, can be labor- and time-intensive. RDS is a network-based sampling design whereby staff initiate the sampling process by purposefully selecting and enrolling a small number of eligible individuals (seeds). These seeds are given a small number of coupons (invitations to the survey) and are asked to randomly select and refer peers in their personal network to the survey. These candidate participants (recruits) present their received coupon to the survey office, enroll, and are then issued coupons as well, hence continuing the peer-recruitment process. RDS data collection includes information about recruits’ personal network size; in analysis, the use of RDS estimators then allows for the generation of weighted, population-level estimates.

Internet-based surveys are appealing as they can be cost-effective and are not restricted by distance, but they typically rely on convenience samples, such as the American Men’s Internet Survey [6]. As internet accessibility is growing and smartphone use is becoming increasingly common, the importance and use of the internet for key population surveillance is growing as well. Web-based RDS surveys hold the promise of weighted estimates for areas as large as the populations are networked but have not become common to date. A few national-level web RDSs among MSM have been conducted in the past, such as in Vietnam [7] and Sweden [8]. We describe here the design, coding, and procedures of a largely automated web RDS system that aimed to replace and fulfill all attributes of a physical, staffed RDS office. A separate publication describes the sampling and survey findings [9].

## Methods

### Survey Goal, Design, and Setting

Our goal was to design and conduct a web RDS among MSM in Thailand. The objectives were 2-fold: to create a ready-to-use (coded) web RDS system and to pilot the feasibility of collecting HIV-related biomarkers through such a sampling design. There was no physical survey office. We developed a website to function as the “RDS office” with all features automated, including eligibility screening, consent, (self-administered) interviewing, e-coupon issuance, peer recruitment training, and electronic compensation. Eligible participants were male, aged 15 years and older, had anal sex with another man in the last 6 months, resided in Thailand, could read Thai, and presented a valid e-coupon (except the seeds). The sampling area comprised all of Thailand; additionally, in Bangkok only (due to logistics), participants who consented were invited to attend select health care clinics for an HIV-related blood draw. There was no specific target sample size; rather, our aim was to sample until at least convergence for salient variables was reached and concluding lessons from the pilot could be drawn.

### Ethical Considerations

The underlying protocol was approved by the Thai Ministry of Public Health Ethics Committee (FWA#0013622) and was determined by the US Centers for Disease Control and Prevention (CDC) to be research with CDC investigators not engaged (Project ID 0900f3eb81afe111). Consent was obtained on the web; the consent language was displayed on the web and could be downloaded. Consent for biomarker collection was obtained separately after interview completion and only for Bangkok residents. We collected personal identifying information (Line [a messenger app] ID, as well as phone number and IP address) to help assess eligibility and facilitate communication (as needed), provide compensation, and prevent duplicate or fraudulent enrollment. Data were anonymized, and the phone numbers and Line IDs were encrypted. Compensation was provided as e-money codes; there were 3 types of compensation, as described in the Compensation section.

### System Architecture and Functionality

#### Overview

We designed the website in modular format to facilitate its reusability for other projects. The website performed all functions typically found in a staffed brick-and-mortar RDS office. The website contained 4 main modules: RDS management, data collection instruments, tracking of project status, and payment. The modular format allowed for increased data security, especially for the payment module, impeding illicit attempts to access our e-coupon code information. This protected the website from unauthorized access to any sensitive data. The website and its database server were hosted on a secured web server installed with both a web application and database firewall, defending it against threats such as SQL injections. Only participants with IP addresses within Thailand could log into our system.

The website was coded in PHP, a general-purpose scripting language; all functions were designed to run on the server side. The system was run in the background of a responsive website developed with HTML5, CSS3, JavaScript, and Bootstrap to give the same browsing experience for participants using either desktop computers, laptops, tablets, or mobile phones. The code is available from the authors upon request. Only the Thai language was used in this survey; participant-facing text was piloted to ensure clear understanding. Graphics and animations were also applied to the website to increase the visual attractiveness of the participant-facing webpages. For the user (ie, RDS participant) no app installation was required. Permanent cookies, also known as persistent cookies, were deployed with participants’ consent and used to check the status of participants’ devices (“not used before,” “currently in use,” “already used in the past”).

#### Data Security

To maximize data security, the system was hosted on a high security web server integrated with numerous cyberthreat countermeasures such as SSL certificates and firewall protection, as well as network and facility (web server) redundancy. The web server also enabled IP filtering to screen for incoming access from outside Thailand (which was disallowed). All personal identifying information was encrypted.

#### Seed Selection and Start of Recruitment

**Figure 1:**
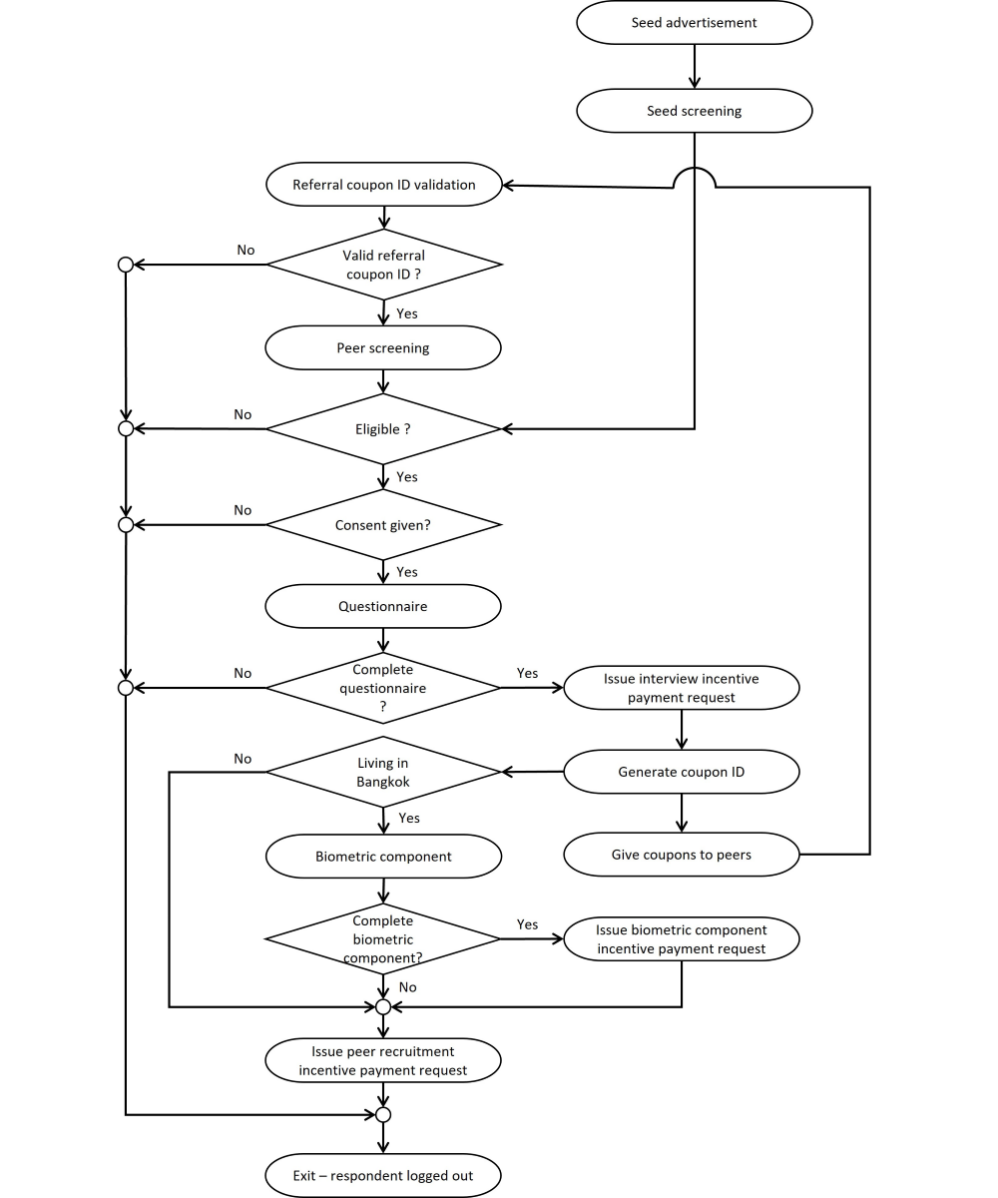
Key events and processes for survey respondents (Kai Noi survey).

Selection of seeds (ie, survey respondents selected by staff who then started the peer-recruitment sampling process) began with advertising in MSM-focused social networks (Figure 1), including the Buddy Station website [10], a Facebook group (Pink Monkey Organization), and the BlueD app; the latter also included livestreaming advertisements. Upon clicking one of these banners, candidate seeds were directed to the survey’s seed recruitment webpage, where project information was shown. Consent to store cookies on their device was obtained. Candidate seeds were asked to submit their mobile phone number to the system and were directed to the seed screening page for the automated eligibility interview. Ineligible candidate seeds were directed to a “thank you” page and exited; eligible candidate seeds provided consent and were enrolled. Thereafter, seeds were transferred to the main interview section. After interview completion, the system sent them an SMS text message with e-coupon codes and a link to the survey for peer referral. Seeds were instructed to send this information to 3 randomly selected peers in their network; e-coupons were then redeemed on a “first come, first served” basis.

#### RDS Management

This module played a major role in the web RDS application. We used participants’ mobile phone numbers as their unique identifiers. To verify the participant’s real existence, a one time password (OTP) was sent to the phone number and had to be re-entered into our system. RDS e-coupons were generated, monitored, and managed. The RDS e-coupon system used uniquely coded e-coupons that allowed recruiters to link with their recruitees. The e-coupon codes used a format of 5 alphanumeric (letters and numbers) digits. Ambiguous letters and numbers (*0*, *o*, *O*, *i*, *I*, *L*, *l*, and *1*) were not used. Codes were generated randomly, out of a pool of 459,165,024 possible combinations of coupon IDs. Once an e-coupon code was generated, it was activated and assigned with the following attributes: *activated*, *date of activation*, *date of expiration*, *cancelled* (ie, invalidated), and *already used*. Each coupon ID could be used only once for enrollment. We issued 3 e-coupons to each recruiter; this number was reduced toward the end of sampling to bring sampling to a controlled halt.

Coupon IDs submitted to the RDS website were checked against the following attributes: whether the e-coupon had indeed been issued, was activated, had not expired, was not canceled, and had not already been used previously. At any time, the RDS project manager and web RDS system administrator could modify all e-coupon attributes, such as activation and expiration dates, cancel (invalidate) coupon IDs, or alter the number of e-coupons to be issued for all recruiters or based on recruiter characteristics, such as residence or age.

#### Eligibility and Consent

The eligibility screening took place directly on our Kai Noi (meaning “small egg” in Thai) survey website, using a short, self-administered interview. All eligibility criteria were based on self-reports. Eligible candidate participants were directed to the consent page; the participant indicated consent by checking the appropriate check box. Respondents refusing consent were directed to a “thank you” page and exited.

#### Main Interview

Following consent, the participant was automatically directed to the main interview, for which we used Lime Survey (Lime Survey) [11], a free and open-source statistical survey web app written in PHP using a MySQL, SQLite, PostgreSQL, or MSSQL database distributed under the GNU General Public License. As web server–based software, Lime Survey provides a web interface to develop and publish surveys, collect response values, create statistics, and export the resulting data to other applications, such as SAS (SAS Institute), Stata (StataCorp), or R (R Foundation for Statistical Computing).

**Figure 2:**
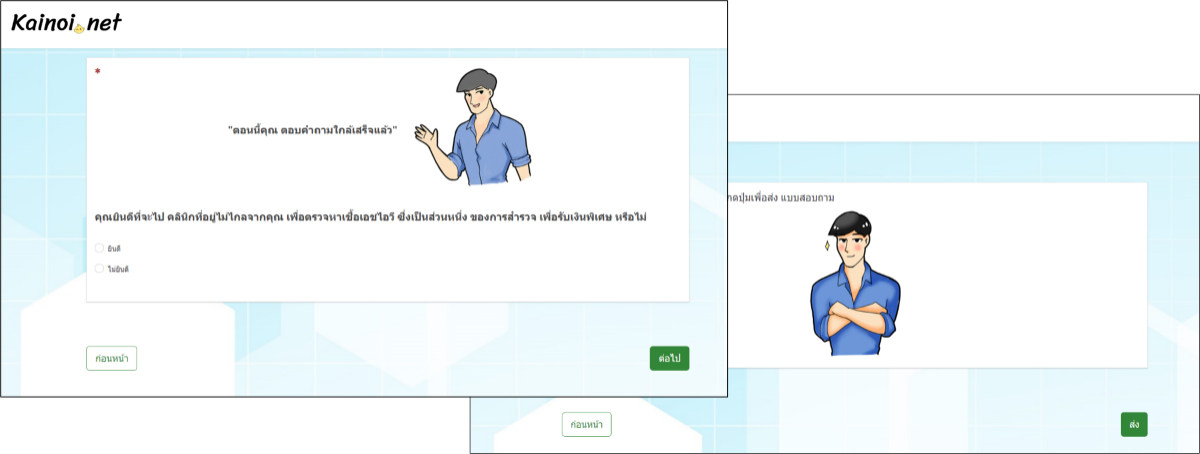
Questionnaire screens on Lime Survey.

To prevent unauthorized access to the participant’s questionnaire, a survey token was autogenerated as an 11-digit number matched with the participant’s own e-coupon code and activated in Lime Survey. The link to Lime Survey was invisible to the participant; any access with an incorrect or inactivated token was ignored by the system. Each interview question was displayed separately; that is, there was no option to scroll down and through the questionnaire. Almost all questions were closed; after selecting a response value, the next question was shown automatically. Programmed skips and required field settings ensured that nonapplicable questions would not be displayed and all applicable questions were answered. The main interview’s database was separate from the RDS management database. Sample questionnaire screens are shown in Figure 2. Interview domains covered essential RDS variables, demographics, internet use, risk behavior, and HIV service uptake. Following completion of the interview, participants were automatically redirected back to the RDS website.

#### Communication With Respondents

Automated communication with the participants, including the use of an OTP, payment, and the issuing of peer referral e-coupons, was based on SMS text messages. True Corporation, Thailand’s second-largest mobile service provider, was chosen as the SMS provider. All SMS messages were sent through requests to its web service. Moreover, only requests sent from specified IP addresses were processed. Participants received the SMS from a sender named Kai Noi (the name of the RDS website). To ensure that the survey’s SMS was not blocked by spam filtering, the sender’s name was registered to whitelists of every mobile service provider in Thailand. The whitelist registration process took about 2 to 4 weeks to become effective prior to the survey start.

#### Compensation

**Figure 3:**
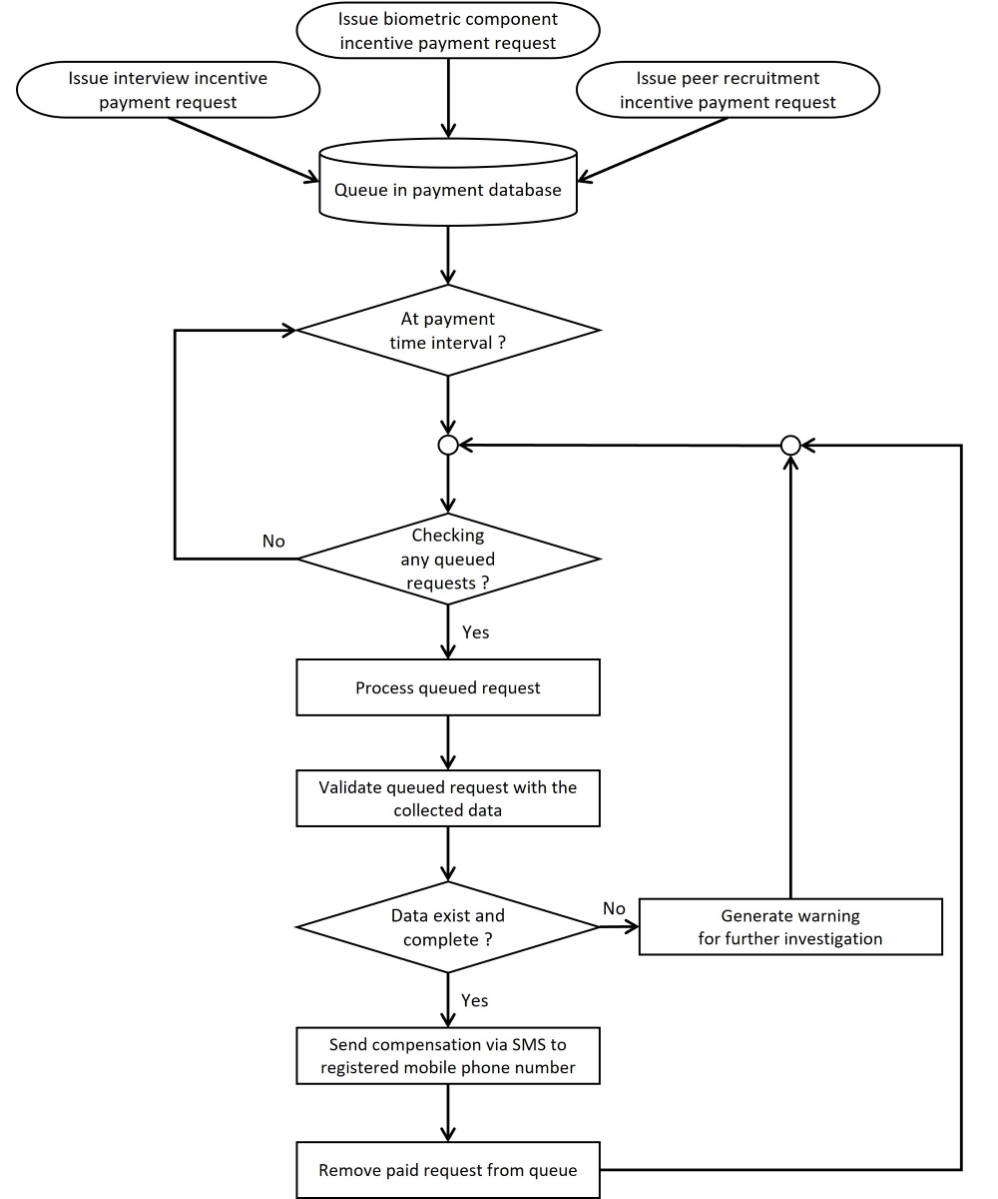
Payment process (Kai Noi survey).

**Figure 4:**
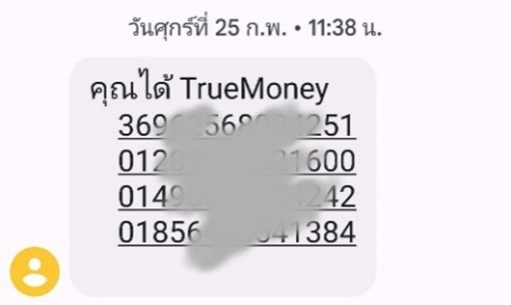
Screenshot of an SMS text message containing codes representing electronic cash sent to a participant’s mobile phone.

Payment was a programmed task executed by the server based on defined events (Figure 3). We used TrueMoney (True Corporation) redemption codes sent by SMS to the participant’s stated mobile phone number. These codes could be redeemed as electronic money to top up the True eWallet. True eWallet is widely used in Thailand, and most convenience stores, restaurants, and online shopping platforms accept True eWallet payments. We applied 3 separate types of payment: for the completed interview (US $9.40), for successful peer recruitment (US $1.60 per referral, defined as a completed interview by the recruitee), and for the blood draw (US $19.40). For peer recruitment, the payment request was calculated based on the number of successful peer referrals and queued after the referral e-coupons had expired. Upon meeting 1 of the 3 payment criteria, the server implemented an internal routine to queue the payment request; payment requests for interview completion were processed every 3 minutes. Peer recruitment payments were processed at 2 AM every day to reduce the load on the web RDS server. The system was designed so that each participant (ie, mobile number) could be paid only once for each of the 3 payment types. Each payment was revalidated before sending the electronic payment by SMS, that is, the system verified that data were indeed collected and that the interview was complete. The system determined the combination of electronic money redemption codes needed to meet the amount to be paid. These electronic money redemption codes were sent with the participant’s registered mobile number as a web request script to the SMS operator’s web service. This then prompted sending the SMS payment to the participant (Figure 4).

#### Project Status Tracking

**Figure 5:**
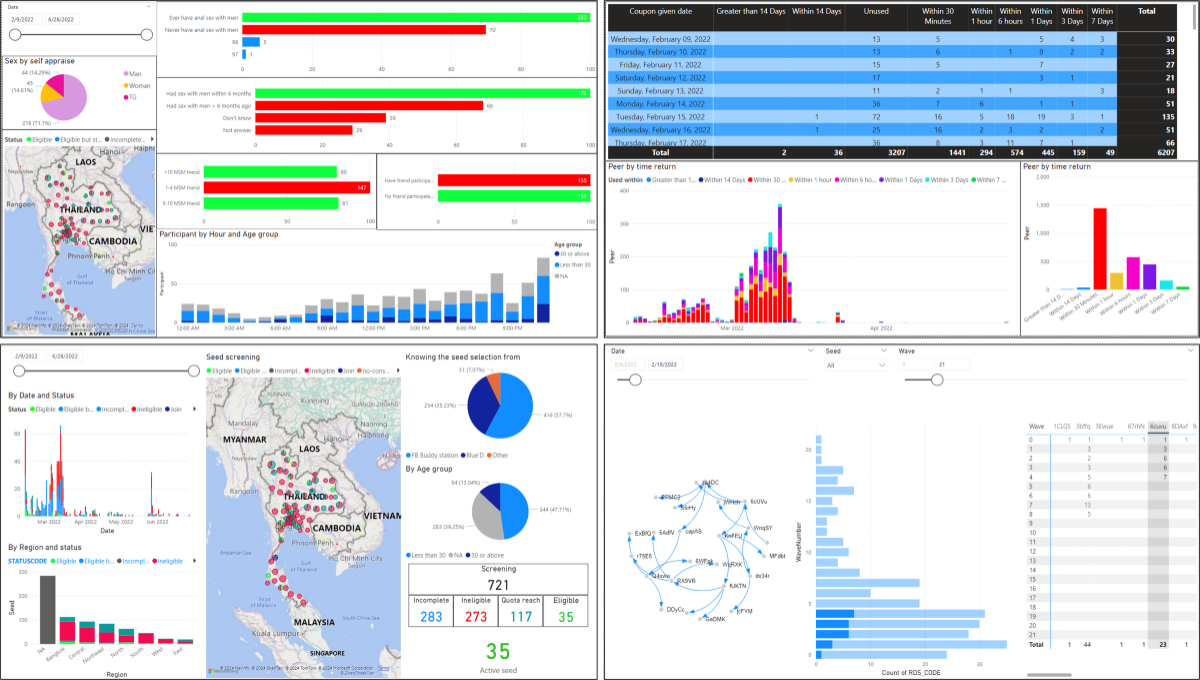
Dashboard screens to track project progress and detect irregularities.

We developed a dashboard (with Microsoft Power BI, as well as with original coding on our project website) to track project progress and detect irregularities (Figure 5). We used a variety of indicators to track sampling, such as peer recruitment, number of participants, sampling speed, and geographic coverage. In addition, our application allowed us to generate RDS network trees that were color-coded to display participant attributes such as age, residential area, and HIV status. Fraud-detecting algorithms based on factors such as time between e-coupon activation and redemption, recruiter and recruitee sharing the same IP address, and multiple uses of the same e-coupon code were also implemented. Any detected suspicious transaction was reported on the project dashboard. We monitored sampling speed and e-coupon uptake, as well as convergence (the point at which the weighted sampling distribution of an estimate stabilizes) and homophily (the tendency for recruits to connect with others with similar characteristics) for select indicators (HIV testing, serostatus knowledge, and condom use at last sex).

#### Peer Recruitment and e-Coupon Management

**Figure 6:**
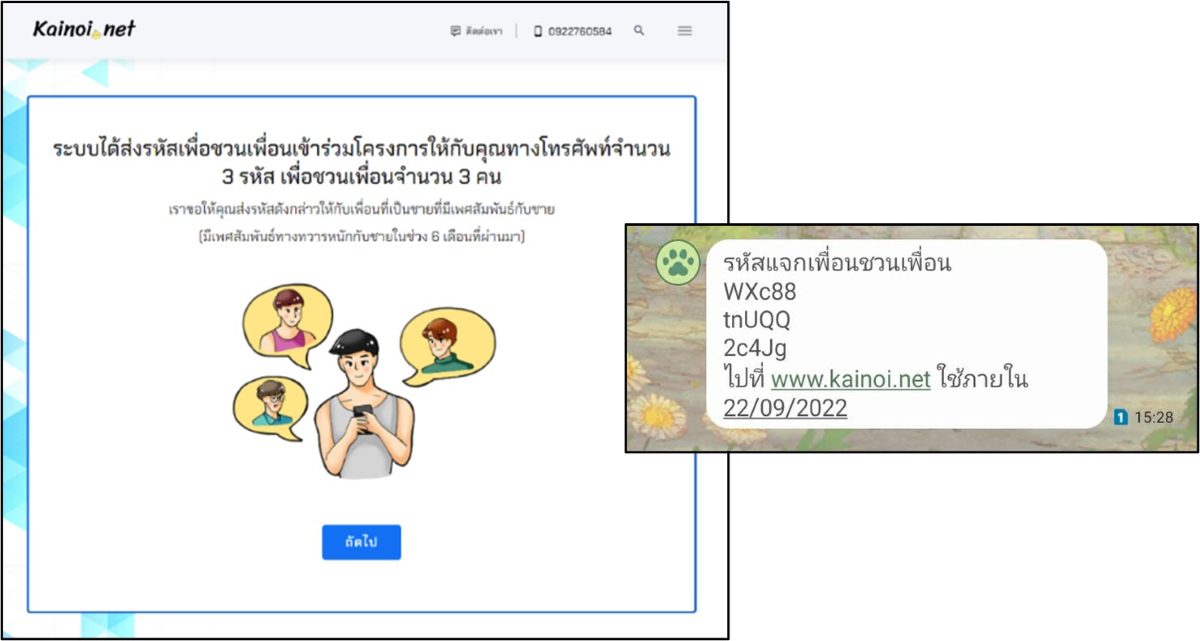
Example of SMS text message with referral coupon codes sent to a participant’s mobile phone.

After interview completion, each participant was sent 3 e-coupon codes alongside the link to the survey website [12] via SMS to their mobile phone number (Figure 6). The participants were instructed to randomly forward the e-coupon codes to their MSM friends and so invite them to join the survey.

#### Recruit Enrollment

For recruits, a separate portal from the one used for seeds was designed for accessing the RDS website. For all recruits, survey enrollment required a valid RDS coupon code and mobile phone number. RDS coupon codes were automatically checked for validity; that is, it was confirmed that the coupon ID number had been issued, had been activated, had not expired, and had not been used already. The mobile phone number was compared against the survey’s database to confirm it had not been used previously in the survey. Candidate participants with both a valid e-coupon and phone number were directed to the screening page; those presenting an invalid e-coupon or phone number were thanked by the system and exited.

Candidate recruits received a brief explanatory message along with the e-coupon and the link to the survey site through which they could access the website, where they were prompted to enter the e-coupon code and their mobile phone number. A short eligibility questionnaire, slightly different from that used for candidate seeds, was displayed. Ineligible candidates were redirected to a “thank you” page without disclosing the exact reason for ineligibility; eligible candidates proceeded to the consent page, followed by the main interview.

#### Biomarker Component

After interview completion, our system automatically selected participants who resided in Bangkok to display an invitation for HIV-related testing. Participants who refused were thanked and exited; our plan was that participants who indicated their willingness for HIV testing would be shown a list of 10 participating clinics in Bangkok and asked to attend one of these clinics in the “coming days” at a time of their choosing. Logistical constraints prevented us from implementing the biomarker component across Thailand. Participants who presented at one of the clinics would then be asked for their registered mobile phone number. The trained clinic staff would then submit the mobile phone number to the system which in turn would send a code to the participant via SMS. The participant was to present the SMS with the code on their phone to the staff to ensure only eligible recruits could reach this stage and to facilitate linking their biomarker data to the correct electronic record. After confirming participant identity (through their mobile phone number), participants would be given an information sheet with the biomarker consent language. Participants refusing consent were to be thanked and exited; verbally consenting participants would undergo a blood draw performed by a licensed nurse. HIV rapid testing, as well as other HIV-related testing, was to be performed according to the testing guidelines in Bangkok at the clinic and off-site laboratories. Clinic staff would enter the test results into the system. An automated request for payment to the participant would be initiated once a blood draw was recorded in the system. These requests were queued in the payment database and processed within 3 minutes. An SMS message with a mobile TrueMoney code worth 500 baht (US $15) would be sent to the participant’s registered mobile phone number. Test results would be merged into the survey’s database for analysis. Test results of clinical utility (eg, HIV status, viral load, and viral hepatitis status) were to be returned to the participant, and treatment or referral offered accordingly.

## Results

### Sampling

During February to June 2022, we initiated sampling with 21 seeds and added 14 more during sampling to increase recruitment (Table 1). A total of 6207 e-coupons were issued and 3000 (45.1%) were redeemed. Sampling reached a maximum of 191 waves, and for select indicators, convergence was reached, though homophily was present to a varying extent. Sampling covered all 6 regions and 75 of 77 provinces; our final sample size was 1615. Sampling across time and space is displayed as a sampling animation in Multimedia Appendix 1. None of the 271 participants residing in Bangkok presented for a blood draw at any of the 10 participating clinics. More details are described in a forthcoming publication by WS.

**Table 1:** Sampling characteristics from e-coupon issuance (n=6207) to the final data set.

Characteristics		e-Coupons, n (%)
Unredeemed		3207 (51.7)
Redeemed		3000 (48.3)
Mobile phone number already registered		464 (7.5)
Screened		2536 (40.9)
Screening incomplete		297 (4.8)
Not eligible		136 (2.2)
Eligible		2103 (33.9)
**Consent provided**		
	No	8 (0.1)
	Yes	2095 (33.8)
**Questionnaire completed**		
	No	64 (1)
	Yes	2031 (32.7)
**Fraud**		
	Yes	317 (5.1)
	No	1714 (27.6)
**Failed attention filter**		
	Yes	99 (1.6)
	No	1615 (26)

Table 2 displays device types and web browsers used to redeem the e-coupon codes on the Kai Noi survey website. Mobile devices accounted for 2090 (69.7%) of the 3000 devices, whereas 910 (30.3%) devices were desktops or laptops. Google Chrome was the most frequently used web browser (n=1664, 83.3%), followed by Safari (n=393, 14.3%) and very few other browsers (n=33, 2.4%).

**Table 2:** e-Coupon (n=3000) redemption by device and browser type.

Category		Redeemed e-coupons, n (%)
**Devices**		
	Mobile	2090 (69.7)
	Desktop or laptop	910 (30.3)
**Web browsers**		
	Google Chrome	1664 (83.3)
	Safari	393 (14.3)
	Other	33 (2.4)

### Recruitment Time

Of the 3000 e-coupons redeemed, 48% (n=1441) were redeemed within 30 minutes and 92% (n=2754) were redeemed within 1 day; only 1.3% (n=38) were redeemed more than a week after issuance (Table 3).

**Table 3:** Time from e-coupon (n=3000) issuance to redemption.

Time	Redemption at each time point, n (%)	Cumulative redemption, n (%)
≤30 minutes	1441 (48)	1441 (48)
>30 minutes to 1 hour	294 (9.8)	1735 (57.8)
>1 hour to 6 hours	574 (19.1)	2309 (77)
>6 hours to 1 day	445 (14.8)	2754 (91.8)
>1 day to 3 days	159 (5.3)	2913 (97.1)
>3 days to 7 days	49 (1.6)	2962 (98.7)
>7 days to 14 days	36 (1.2)	2998 (99.9)
>14 days	2 (0.1)	3000 (100)

### Data Collection and Quality

The median duration of the main interview was 2.1 (IQR 1.7-2.8) minutes. There were no missing interview data. The attention filter question was not passed successfully by 99 (1.6%) participants; that is, these individuals did not select the answer that they were asked to select. We removed their interview data but retained the remaining survey records in the data set (for eligibility and personal network size) and allowed their referred peers to fully remain in the survey’s database. We also examined the interview data for signs of straightlining, that is, for a set of 9 adjacent questions that were not subject to any programmed skips, and scanned the survey records for strings of identical response values for these 9 questions. One such record was identified as showing straightlining; that record belonged to a fraudulent survey enrollment (see below).

### Fraud

Multiple fraudulent enrollments (n=318, including n=1 seed and n=317 recruits) were detected. Fraud was initially suspected by staff when a survey participant called the project phone number to inquire whether an incentive payment had been paid yet. These calls were received multiple times and were related to different enrollments but were seemingly made by the same person. When our staff called back the listed mobile phone numbers, we found that the phone numbers’ owners had never participated in the Kai Noi survey. Conversations with these persons indicated that their phone numbers were “rented out” to someone on the internet and that they were asked to forward all SMS messages received from our RDS website to the imposter in exchange for US $0.60-$1.60. The fraudster apparently also cleared the cookies on their device after each interview completion or used incognito mode on the web browser to bypass the cookie check. We were unable to determine how many real persons were behind these fraudulent enrollments. After detecting these fraudulent enrollments on day 33 after the survey start, we disabled all referral e-coupons related to them. Subsequently, for all recruits, we changed the timing of paying compensation for enrollment from the original 3 minutes after survey completion to 3 days, thereby making fraud less attractive. Following these changes, we no longer detected signs of fraud. All data related to fraudulent enrollments were excluded from analysis.

## Discussion

In this paper, we describe the architecture and real-world functionality of a web-based RDS among MSM in Thailand. Our in-house code recreated all RDS features typically found in a brick-and-mortar, staffed RDS office and executed essentially all survey procedures using algorithms. Our staff presence was limited to monitoring survey events on the web and answering the occasional call or text from respondents with questions. Compared to a physical RDS, a web RDS such as Kai Noi promises significant advances and efficiencies. However, we also faced substantial challenges, including fraud and zero biomarker uptake.

A key strength of our web-based RDS was that the sampling area comprised the entire country, in contrast to physical RDSs, which are typically limited in geographic scale as they require respondents to travel to the survey office. The RDS design facilitated a weighted analysis using, for example, the *RDS Analyst* R package to adjust for differences in sampling [13], including for any over- or undersampling of particular survey regions; nevertheless, our sample’s spatial distribution did not show extreme differences compared to the general population’s distribution across Thailand’s 6 large regions (data not shown). Our automated survey design also allowed for data collection at any time of day with minimum staff involvement. Of note, our survey was launched during the peak period of COVID-19 transmission in Thailand. The number of persons testing for HIV at the same 10 sites as in our survey decreased from 82,054 in 2020 to 60,124 in 2022 during the fifth wave of the Omicron strain of COVID-19 [14].

Fraud in surveys using incentives is not uncommon and was previously observed in web-based RDSs [15]. In our survey, we believe that some 15% of survey enrollments were fraudulent. The lack of physical staff-respondent interaction likely increased the risk of fraud, and automated survey execution facilitated such fraud on a larger scale. The use of other persons’ mobile phone numbers was the principal means fraudsters used to repeatedly enroll in our survey. We believe that our measures of invalidating all e-coupons related to fraudulent enrollments, delaying e-coupon activation, and delaying compensation payout may have stopped the scam and significantly decreased the motivation to commit fraud. Still, additional measures should be used in future surveys, such as greater use of the enrolled mobile phone number, not just for enrollment, but perhaps also to verify select interview responses, solicit or resolicit them, or renew phone-based authentication during the interview with a short response time window. Having more than one phone number poses additional potential for fraud [16] that may be (partially) detected by identifying identical recruiter-recruit interview response patterns; however, the absence of such suspect patterns does not exclude the risk of fraud. Survey investigators may choose to probe for additional phone numbers during the beginning of the enrollment process; this may help minimize duplicate enrollment to some extent. Our banner ads included information about compensation, which may have prompted the interest of a fraudulent seed respondent. Consideration should also be given to start web-based RDSs only with seeds known to and trusted by investigators, if feasible. A more stringent measure may include predefined sampling stops (no issuance of coupons) once a given recruitment chain reaches a prespecified length, although this may necessitate repeated “reseeding,” reduce total sample size, and impact convergence and homophily.

Of note are some of the interview characteristics. The mean duration of the main interview was short, and yet most respondents successfully passed the attention filter question; as well, there was very little sign of straightlining. We have previously discussed the observed lack of biomarker uptake [9].

Our code was developed in open-source environments and is available upon request from the investigators; while its modular concept design allows for easy adaptation, it will require further adaptation to each survey’s unique setting and characteristics. Survey staff may engage computer programmers to create, modify, and administer the web-RDS code as needed in real time. Further, a smooth survey experience calls for web server hosting with reasonable bandwidth, reliability, scalability, and security. Cloud web hosting may be considered in settings lacking sufficient internet infrastructure.

This survey adds to the small but growing list of web-based surveys using network-based sampling. Developing the software code and addressing the risk of fraud are the 2 main hurdles. Making such code available to other developers for further improvement may help accelerate the creation of standing virtual RDS offices that can initiate sampling at shorter notice with less effort and at lower cost compared to physical survey efforts. However, the risk of fraud will remain and require digital preventative measures and proactive monitoring. While fraud may not be fully eliminated, a combined set of measures may minimize its risk and scale.

## Appendix

Multimedia Appendix 1 Sampling animation.

## Abbreviations

CDC: US Centers for Disease Control and Prevention
MSM: men who have sex with men
OTP: one time password
RDS: respondent-driven sampling
